# Besserung der Hautmanifestationen des SLE mit monogenem Komplementdefekt durch Typ‐I‐Interferonblockade mit Anifrolumab

**DOI:** 10.1111/ddg.15806_g

**Published:** 2025-10-23

**Authors:** Julian Steininger, Christine Wolf, Min Ae Lee‐Kirsch, Claudia Günther

**Affiliations:** ^1^ Klinik und Poliklinik für Dermatologie Universitätsklinikum Carl Gustav Carus Technische Universität Dresden; ^2^ Klinik und Poliklinik für Kinder‐ und Jugendmedizin Universitätsklinikum Carl Gustav Carus Technische Universität Dresden; ^3^ UniversitätsCentrum für Seltene Erkrankungen (USE) Universitätsklinikum Carl Gustav Carus Technische Universität Dresden; ^4^ Deutsches Zentrum für Kinder‐ und Jugendgesundheit (DZKJ) Partnerstandort Leipzig/Dresden; ^5^ Universitäts‐Hautklinik, Universitätsklinik Tübingen Eberhard Karls Universität Tübingen

**Keywords:** CLASI, C1q‐Defizienz, Interferon, kutaner Lupus, Lupus erythematodes, SLE, CLASI, C1q deficiency, cutaneous lupus, interferon, Lupus erythematosus, SLE

Sehr geehrte Herausgeber,

Wir berichten über die vollständige Remission (CR) diskoider kutaner und chilblainartiger Lupusläsionen bei einem 24‐jährigen Mann mit biallelischer *C1QC*‐Mutation^1^ nach Behandlung mit dem Interferon‐α/β‐Rezeptor (IFNAR)‐Antagonisten Anifrolumab.

Monogene Veränderungen im *C1Q* (C1QDef) sind äußerst selten und wurden bislang in nur 74 Fällen beschrieben.^2^ Diese Veränderungen sind stark mit einer erhöhten Expression von Typ‐I‐Interferon (IFN) sowie von Typ‐I‐IFN‐induzierten Genen (ISG) assoziiert und induzieren Symptome eines systemischen Lupus erythematodes (SLE). Die Symptome bei Patienten mit C1QDef manifestieren sich in der Regel bereits in der Kindheit und zeigen außerdem eine eher reduzierte Ansprechrate auf gängige SLE‐Therapien. Infolgedessen wurden in Fallberichten Therapieversuche mit hohem Komplikationsrisiko, wie beispielsweise hämatopoetische Stammzelltransplantationen, beschrieben. Zusätzlich zur signifikanten Morbidität ist C1QDef auch mit einer erhöhten Mortalität assoziiert.^3^

Unser Patient zeigte bereits im Alter von 4 Jahren (10/2003) diskoide Hautmanifestationen, weshalb eine Therapie mit Hydroxychloroquin eingeleitet wurde (Abbildung 1). Im April 2004 traten Proteinurie, erhöhte Rheumafaktoren sowie antinukleäre, Anti‐Kardiolipin‐ und Anti‐SSA‐Antikörper auf, was insgesamt auf eine systemische Beteiligung, im Sinne eines SLE, hinwies. Zwei Jahre später entwickelten sich undulierend auftretende Schmerzen und Schwellungen in den Ellbogengelenken. Zudem wurden grenzwertig hohe Anti‐Doppelstrang‐DNA‐Antikörper nachgewiesen, weshalb eine Therapie mit Azathioprin und Prednisolon eingeleitet wurde. Aufgrund einer Nierenschädigung mit Makrohämaturie und Proteinurie (12/2008) erfolgte eine Umstellung zu Mycophenolat (MMF) in Kombination mit Hydroxychloroquin.

**ABBILDUNG 1:**
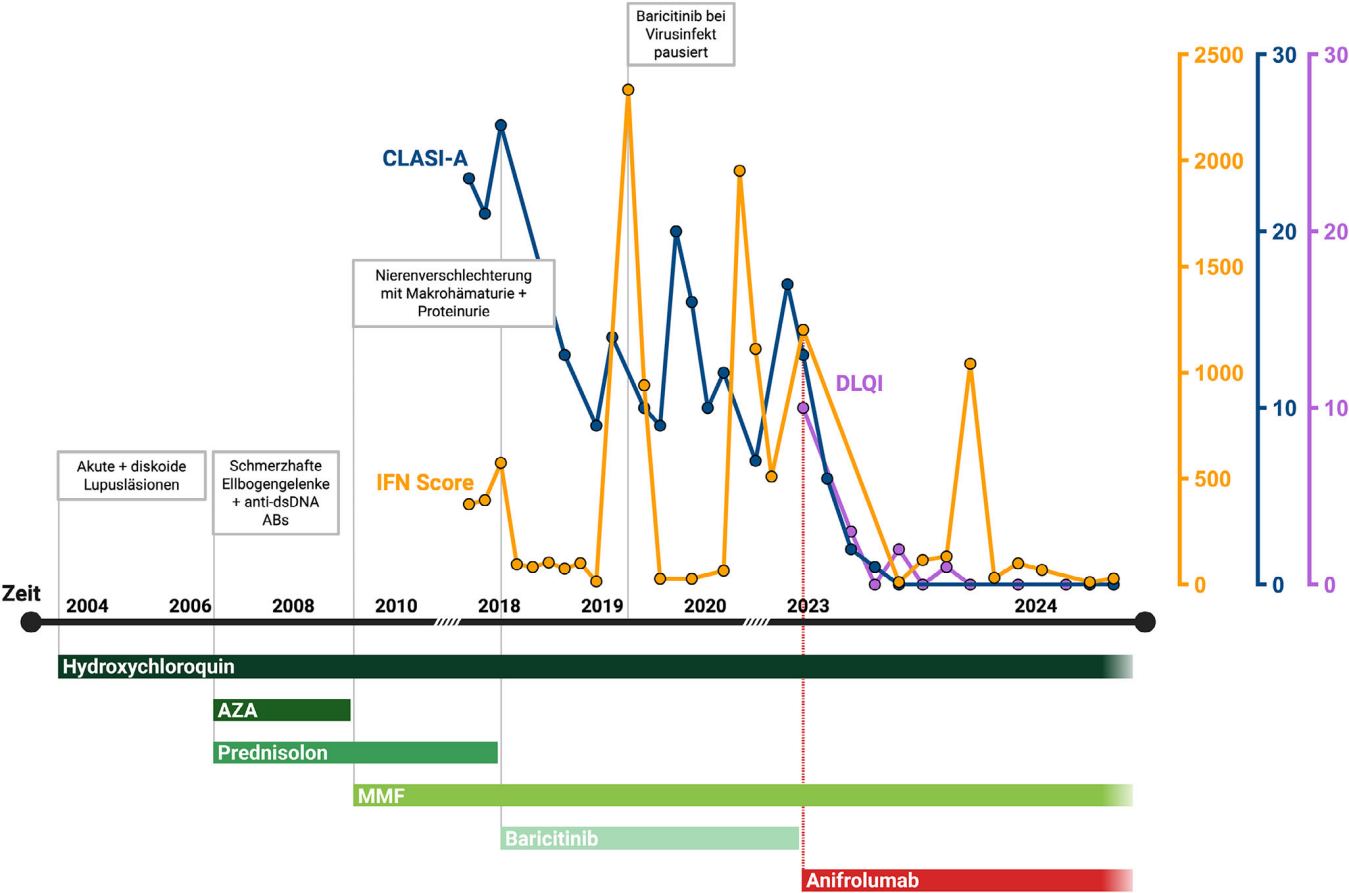
Verlaufsdarstellung eines Patienten mit biallelischer *C1QC*‐Mutation. Zeitlicher Verlauf der klinischen Präsentation sowie der eingesetzten Medikation. Die orangefarbene Linie zeigt den Interferon (IFN)‐Score des Patienten. Die blaue Linie stellt den Aktivitätswert des CLASI (CLASI‐A) dar. Die violette Linie gibt den Wert im *Dermatology Life Quality Index* (DLQI) an. Der IFN‐Score wurde durch Messung der mRNA‐Expression von sieben ISG (IFI27, IFI44, IFI44L, IFIT1, ISG15, SIGLEC1 und RSAD2) bestimmt, normiert auf GAPDH und HPRT1, und im peripheren mononukleären Blutzellkompartiment mit einer gesunden Kontrollkohorte verglichen.¹ Abk.: AZA, Azathioprin; MMF, Mycophenolatmofetil

Ab 2015 erforderten wiederholte SLE‐Schübe, einschließlich progredienter kutaner (CLE) Beschwerden (Abbildung 2), eine monatliche Stoßtherapie mit Prednisolon. Infolgedessen stellte sich der Patient im Februar 2018 erstmals in unserer Klinik vor, wo eine homozygote Mutation im *C1QC*‐Gen (c.205C>T, p.Arg69Ter) diagnostiziert wurde. Aufgrund der unzureichenden Krankheitskontrolle erweiterten wir die immunmodulierende Medikation um dem Januskinase (JAK)‐Inhibitor Baricitinib, was insgesamt zu einer deutlichen Besserung der Symptome führte.^1^ Allerdings persistierten insbesondere im Gesicht diskoide Plaques, und die Werte des *Cutaneous Lupus Erythematosus Disease Area and Severity Index Activity* (CLASI‐A)^4^ normalisierten sich nicht vollständig (Abbildungen 1, 2). Interessanterweise zeigte sich ein signifikanter Abfall der Typ‐I‐IFN‐Signatur im Blut, ohne dass jemals eine CR des CLE erreicht werden konnte. Aufgrund des unzureichenden klinischen Ansprechens wurden die JAK‐Inhibitoren abgesetzt und eine neu verfügbare Therapie mit Anifrolumab (300 mg alle 4 Wochen) eingeleitet.

**ABBILDUNG 2:**
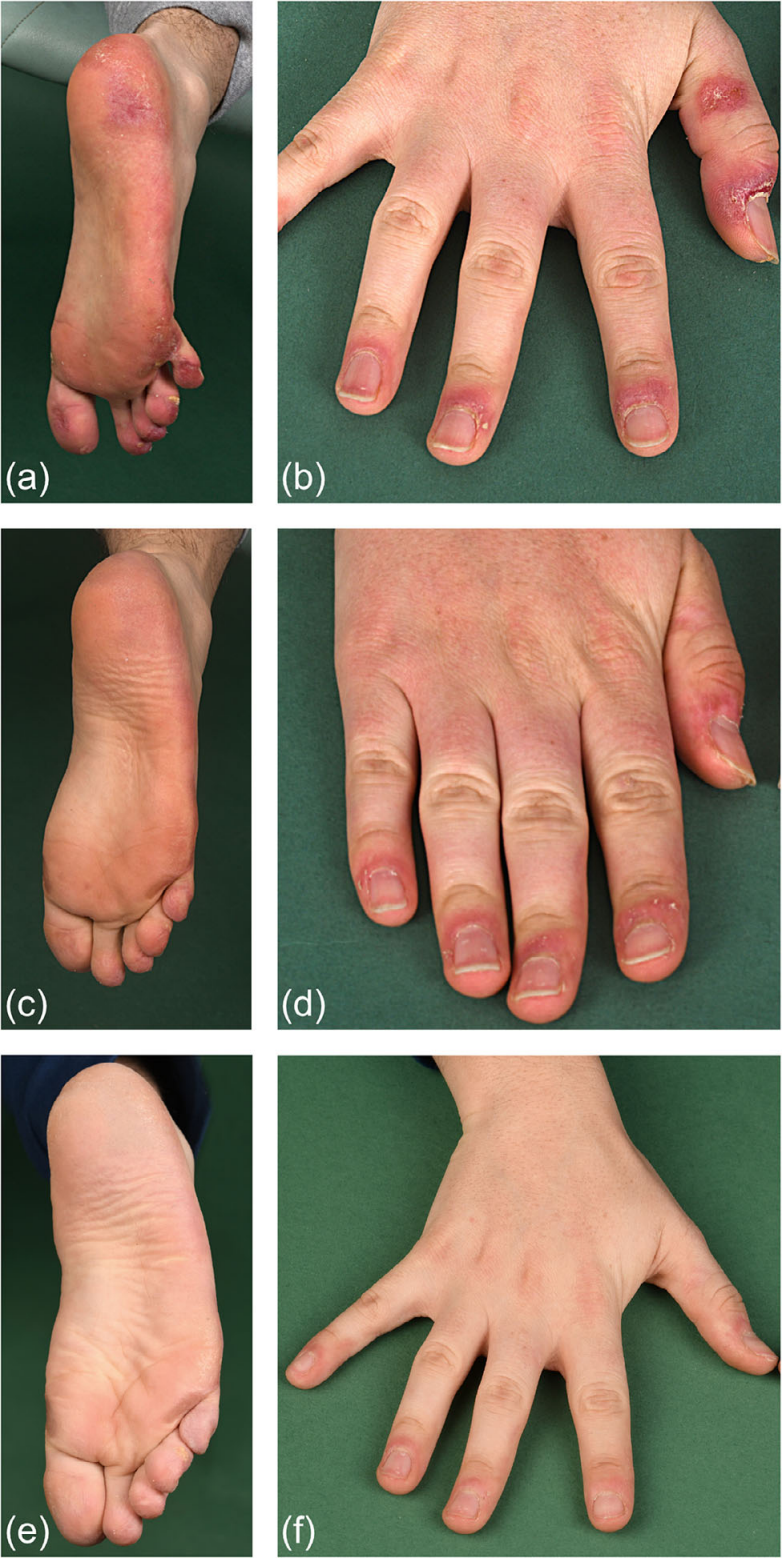
Krankheitsbesserung bei einem Patienten mit biallelischer *C1QC*‐Mutation unter Behandlung mit dem Interferon‐α/β‐Rezeptor (IFNAR)‐Antagonisten Anifrolumab. (a, b) Unscharf begrenzte livide erythematöse Plaques an den Füßen mit Schmerzen beim Gehen; akrale erythematöse Erosionen und Ulzerationen an den Händen. (c, d) Drei Monate nach Beginn der Therapie mit Anifrolumab mit Besserung der Hautveränderungen an Füßen und Händen. (e, f) Nahezu vollständige Abheilung der Hautläsionen an Füßen und Händen nach einjähriger Therapie mit Anifrolumab.

Anifrolumab ist ein monoklonaler IgG1κ‐Antikörper, der die Bindung von Typ‐I‐IFNen an ihren gemeinsamen Rezeptor hemmt. Studien konnten seine Wirksamkeit bei der Behandlung von CLE‐Läsionen belegen.^5^, ^6^ Zudem konnte das Medikament eine besonders starke Wirkung bei Patienten mit erhöhtem Typ‐I‐IFN zeigen^7^, ^8^ und IFN‐Werte bei SLE‐Patienten signifikant reduzieren.^9^

Bereits nach der ersten Infusion von Anifrolumab trat eine rasche klinische Besserung ein: Zuvor therapieresistente Hautläsionen verbesserten sich innerhalb eines Monats um 50% (gemessen an den CLASI‐A‐Werten) und verschwanden nach 12 Monaten nahezu vollständig (Abbildung 1, 2). Zudem sprachen die vaskulitischen und chilblainartigen Läsionen an den Extremitäten auf die neu eingeleitete Therapie an, was die bislang selten berichtete Wirksamkeit von Anifrolumab bei Chilblain‐Lupus unterstreicht.^10^ Begleitend verbesserte sich auch die Lebensqualität des Patienten (Abbildung 1).

Der Patient zeigte keine schweren Nebenwirkungen, berichtete jedoch von einer Zunahme leichter Atemwegsinfektionen. Ebenfalls entwickelte sich erstmalig eine Herpes‐zoster‐Infektion im Thoraxbereich, weshalb die Behandlungsintervalle auf 6 Wochen gestreckt wurden. Hierunter traten bereits nach 2 Monaten wiederholt CLE‐Schübe auf, sodass die Intervalle wiederum auf 4 Wochen verkürzt wurden.

Die Kombination von Anifrolumab mit der Basistherapie aus Hydroxychloroquin und MMF führte zu einer überlegenen CLE‐Kontrolle im Vergleich zu Baricitinib (medianer CLASI‐A Wert Baricitinib 13,00, medianer CLASI‐A‐Wert Anifrolumab 1,00; p = 0,0009).^6^, ^7^, ^11^, ^12^ Konkordant mit unseren Ergebnissen berichteten auch Triaille et al. von einer unzureichenden Krankheitskontrolle bei C1QDef‐Patienten unter JAK‐Inhibition.^3^ C1QDef‐Patienten weisen ähnlich hohe Typ‐I‐IFN‐Score‐Werte wie Patienten mit klassischen Interferonopathien auf, was auf eine pathogenetische Hauptbeteiligung des Typ‐I‐IFN‐Signalweges hindeutet. JAK‐Inhibitoren beeinflussen wiederum mehrere Signalwege, da JAK an der Signaltransduktion verschiedener Zytokine beteiligt sind. Demzufolge könnte die Verwendung von JAK‐Inhibitoren eine breitere, aber potenziell weniger vollständige Blockade des Typ‐I‐IFN‐Weges bieten. Im Gegenzug vermag Anifrolumab durch eine direkte IFNAR‐Inhibition möglicherweise eine gezieltere und effektiviere Unterbrechung des hauptverantwortlichen Signalweges zu erzielen.

Es gibt aktuell keine Head‐to‐Head‐Studien zum Vergleich von JAK‐Inhibitoren und Anifrolumab. Der intraindividuelle Therapieverlauf bei diesem Patienten legt nahe, dass Anifrolumab eine überlegene pharmakologische Wirksamkeit bei kutanem CLE im Kontext eines monogenen SLE besitzen könnte. Interessanterweise spiegelt sich das verbesserte klinische Ansprechen nicht im Typ‐I‐IFN‐Score im Blut wider, der unter sowohl Baricitinib als auch Anifrolumab in vergleichbarem Ausmaß abnahm. Demnach ist es wichtig, CLE‐Manifestationen in klinischen SLE‐Studien durch verifizierte klinische Scores zu dokumentieren und ihre Korrelation mit den ISG‐Werten im Blut weiter zu validieren.

## DANKSAGUNG

Open access Veröffentlichung ermöglicht und organisiert durch Projekt DEAL.

## FINANZIERUNG

Diese Arbeit wurde von der *Deutschen Forschungsgemeinschaft* (DFG), Grant TRR237 369799452/404458960 an C.G. unterstützt. M.L.‐K. wird durch DFG‐Fördermittel CRC237 369799452/B21, CRC237 369799452/A11, CRC369 501752319/C06 sowie durch Fördermittel des *Bundesministeriums für Bildung und Forschung* (BMBF) 01GM2206C (GAIN) und 01GL2405H (DZKJ) unterstützt. C.W. wird durch den DFG‐Zuschuss CRC237 369799452/A06 unterstützt.

## INTERESSENKONFLIKT

C.G. erhielt Honorare für wissenschaftliche Vorträge, Gremien und Forschungsfinanzierungen von Astra Zeneca, GSK, Boehringer, Almirall und Janssen. Die anderen Autoren erklären, dass die Forschung in Abwesenheit kommerzieller oder finanzieller Beziehungen durchgeführt wurde, welche als potenzieller Interessenkonflikt ausgelegt werden könnten.
